# Effects of Working Memory Capacity and Tasks in Processing L2 Complex Sentence: Evidence from Chinese-English Bilinguals

**DOI:** 10.3389/fpsyg.2017.00595

**Published:** 2017-04-20

**Authors:** Huixia Zhou, Sonja Rossi, Baoguo Chen

**Affiliations:** ^1^Key Laboratory of Behavioral Science, Institute of Psychology, Chinese Academy of SciencesBeijing, China; ^2^Beijing Key Laboratory of Applied Experimental Psychology, School of Psychology, Beijing Normal UniversityBeijing, China; ^3^Clinic for Hearing-, Speech- and Voice-Disorders, Medical University of InnsbruckInnsbruck, Austria

**Keywords:** working memory capacity, second language, grammatical judgment, translation, sentence processing

## Abstract

Two experiments aimed at investigating how working memory capacity (WMC) related to processing wh-extractions in both a grammatical judgment and a translation task by using the Operation Span task. A self-paced paradigm was used to collect response times and accuracy rates. In Experiment 1, results showed that high WMC was related to faster grammatical judgment of the critical region in subject- and object-extractions. In Experiment 2, high WMC was only related to high accuracy in translating wh-extractions. These results indicate that individual differences in WMC play a certain role during L2 sentence processing, and experimental tasks can modulate this effect.

## Introduction

Working memory, as a system for storing and processing information temporarily when finishing higher-level cognitive tasks, like language understanding, has been the focus of extensive research in cognitive psychology and psycholinguistics (Just and Carpenter, 1992; MacDonald et al., 1992; Baddeley and Logie, 1999; Kim and Christianson, 2007; Rodríguez, 2008; Swets et al., 2008). Differences in working memory (WM) have been related to performance in cognition and language processing in experimental, neuro-cognitive, and individual differences research (Miyake and Shah, 1999; Oberauer et al., 2003; Dallas et al., 2013; Hopp, 2013).

The Constrained Capacity Model (Just and Carpenter, 1992) has been proposed to explicate individual differences of working memory in language comprehension. According to this model, there exists a general verbal WM system with a limited capacity. In addition, information storing and processing consumes the restricted cognitive resources of WM. If the application of WM resources approaches its limit, there will exist a trade-off between the storage and processing component of WM so as to coordinate between the concurrent processes. Such an allocation of cognitive resources is sensitive to specific tasks employed and would be influenced by strategies used by different individuals.

Studies in first language (L1) have shown that working memory capacity (WMC) influences sentence processing, demonstrating that participants with high WMC have advantages in sentence processing compared with those with low WMC (Just and Carpenter, 1992; MacDonald et al., 1992; Kim and Christianson, 2007; Chen et al., 2008; Swets et al., 2008). Comparatively speaking, memory requirements are augmented when processing in a second language (L2), particularly when it is acquired later (Miyake and Friedman, 1998; Michael and Gollan, 2005). The augmented memory requirements on processing in an L2 would successively limit L2 learners' capability to fully catch sight of some syntactic nuances which are automatically finished in L1 processing (Kotz and Elston-Güttler, 2004; Silverberg and Samuel, 2004). Therefore, it is important to investigate the effects of WMC in L2 processing.

Many studies have explored the role of WMC in L2 syntactic processing, but the results are inconsistent. Some demonstrate that WMC is not an essential factor in L2 learners' ability to process complex syntactic structures (Juffs, 2004, 2005, 2006; Rodríguez, 2008; Hopp, 2013). For example, Juffs (2004, 2005, 2006) explored whether WMC could explain the performance differences of L2 learners in grammatical judgment of structures with a very high processing load. The WMC was measured in both L1 and L2. However, no reliable evidence of WMC was found. Rodríguez (2008) examined the relationship between WMC and three complicated structures with a high processing load in high fluent Spanish-English bilinguals. A Spanish reading span task (L1) was administered to the participants, but the results showed that there was no influence of WMC in a reading comprehension task. Hopp (2013) investigated whether L2 WMC affects the German-English bilinguals' processing of object-subject ambiguities, and no reliable effects of WMC were found in a reading comprehension task.

However, some other studies demonstrate that WMC can indeed be a potential predictor for syntactic processing in L2 learners (Sagarra, 2007; Havik et al., 2009; Dussias and Piñar, 2010; Sagarra and Herschensohn, 2010). Dussias and Piñar (2010) examined processing of wh-extractions in Chinese-English bilinguals, and they also manipulated the sentence plausibility. High-WMC but not low-WMC learners were found to be sensitive to subject-object processing asymmetries and sentence plausibility in a grammatical judgment task. Dussias and Piñar used a version of the Waters and Caplan (1996) Reading Span test, which was created in English as an L2. Havik et al. (2009) also reported WMC effects in processing subject-object relative clause ambiguities by German learners of Dutch. The results revealed that only high-WMC L2 learners (measured in both L1 and L2) slowed down in processing object-relative clauses rather than subject-relative clauses in a reading comprehension task. Sagarra (2007) examined whether low-proficient Spanish learners' WMC (measured in L1) affects their sensitivity to gender agreement violations. Higher WMC learners are more accurate than those with lower WMC in processing sentences with gender agreement violations in a grammatical judgment task. Later, Sagarra and Herschensohn (2010) showed that the L1 WMC of their intermediate Spanish learners correlated positively not only with their reading times but also with their grammatical judgment accuracies in sentences containing gender agreement violations.

The findings concerning WMC's role in L2 sentence processing are inconsistent at present. The reasons for such inconsistency may be manifold. By analyzing the previous studies concerning WMC and L2 sentence processing, we found that most of them used L1 and L2 reading span tasks to measure WMC. A large body of research on the psychology of reading and comprehension were founded on the reading span measure of Daneman and Carpenter (1980) and Conway et al. (2005), which was demonstrated to correlate with reading comprehension and Verbal Scholastic Aptitude Test (VSAT) performance to a great extent (Daneman and Carpenter, 1980). The domain-specific skills in verbal ability were found to support better memory for the previously read sentences (Ericsson and Kintsch, 1995; Daneman and Merikle, 1996; Baddeley, 2003) rather than the genuine WMC of participants (Sanchez et al., 2010; Tse and Altarriba, 2014). In addition, some studies found that WMC measured in the L2 may interact with language proficiency (Service et al., 2002; Omaki, 2005; Van den Noort et al., 2006; Coughlin and Tremblay, 2012), thus there may exist the problem of confounding effects between WMC and L2 proficiency.

Some people have proposed that the reading span task is just one of the potential measurements for measuring WMC (Lewandowsky et al., 2010). To lessen the influence of language-specific variance, a series of heterogeneous indexes to assess a general construct such as WMC should be used (Wittmann, 1988; Oberauer, 2005). Compared with the reading span task, the operational span (OSPAN) task which requires participants to solve a series of math operations while trying to remember a set of unrelated letters (Unsworth et al., 2005), has a much lower language requirement, thus making it more likely to reflect L2 learners' genuine WMC (Sanchez et al., 2010). It can eliminate the potential influences of verbal proficiency by requiring the participants to recollect unrelated letters rather than sentence-final words. Compared to extensive involvement of the experimenter in the reading span task, the OSPAN is less prone to human error and variation in performance due to differential instructions as well (Atkins et al., 2014). Zhou et al. (2016) adopted both the L2 reading span task and the OSPAN task to explore whether WMC affects the processing of subject- and object-extraction by Chinese learners of English. Their results demonstrated that L2 reading span has no effect on processing either the subject-extractions or the object-extractions, while, the OSPAN was related to the processing of both kinds of wh-extractions. Therefore, OSPAN seems to be more suitable for investigating the effects of WMC while processing wh-extractions of Chinese-English bilinguals. So, the first purpose of the present study was to further address whether WMC measured by the operation span task would influence L2 sentence processing.

Havik et al. (2009) showed that task demands may also modulate the effects of WMC. According to previous studies (Juffs, 2004, 2005, 2006; Sagarra, 2007; Rodríguez, 2008; Havik et al., 2009; Dussias and Piñar, 2010; Sagarra and Herschensohn, 2010; Dallas et al., 2013; Hopp, 2013), we infer that differences in task-specific demands across studies may be another possible reason for inconsistent results concerning effects of WMC on processing L2 syntactic information. Task-specific demands mean that different studies adopted different tasks. Some of them put emphasis on the processing of semantics, while others emphasized the processing of syntax. Therefore, the experimental results may be influenced by different tasks. However, only a few studies investigated whether or not bilinguals' real-time processing is influenced by variations in WMC, which is furthermore assumed to rely on what kind of task subjects are required to complete in processing the experimental items (Juffs and Harrington, 2011). For instance, by using a self-paced reading task, Havik et al. (2009) examined how advanced German L2 learners of Dutch process the temporarily ambiguous subject- and object-relative clauses. Differences in WMC (assessed by standardized reading span tests measured in both L1 and L2) among the learners did not influence the processing of sentences with temporary ambiguity. In a similar experiment, participants were asked to make a truth-value judgment about the argument roles of the nouns with ambiguity after every experimental item. Results showed that when the number of the auxiliary verb compelled an interpretation of the dispreferred object-first interpretation, high-WMC learners slowed down following disambiguation of the relative clause just as the Dutch natives did. On the other hand, low-WMC learners processed both types of sentences similarly, demonstrating no disadvantage for processing the dispreferred object-relatives. Thus, only the high-WMC learners showed processing patterns similar to native speakers, which was the case only when L2 learners' attention was directed by the task to the experimental manipulation.

In a self-paced reading experiment, Williams (2006) found that WMC influences the real-time processing of wh-extractions like sentence (1) in L2 learners of English with different native languages.

1. Which girl (river) did the man push the bike into late last night?

Participants were required to make a “stop-making-sense” decision when reading such sentences. After reading the whole sentence, they completed an additional memory probe task, in which they were asked to use a word which comes along in the previous sentence to complete another sentence. The author observed that only learners with high WMC, as assessed by the probe task, performed like native speakers. Especially, they can use plausibility information (making “stop-making-sense” reactions) when the sentence itself was presented, in a similar way to native speakers. Compared to the “stop-making-sense” responses of the plausible sentences (which river-push), their responses occurred earlier for sentences in which the displaced wh-phrase was an implausible object for the verb (which girl-push). This response pattern was different from low-WMC learners, who applied the plausibility information later in the sentence.

Therefore, high-WMC participants performed like native speakers only when they had to undertake an experimental task asking them to monitor the sentence meaning for semantic plausibility or truth value judgment (Dussias and Piñar, 2010).

The studies (Williams, 2006; Havik et al., 2009) mentioned above all explored within-language reading tasks (King and Just, 1991). Also using a self-paced reading paradigm, Macizo and Bajo (2006) examined whether between-language reading (reading for translation) and within-language reading (reading for repetition) involve the same processes in translators and bilinguals. Ambiguous sentences, such as (2) containing a homograph, and control sentences, such as (3), were read by professional translators and Spanish-English bilinguals.

(2) Durante toda la sobremesa charlamos sobre el/presente/y estuvimos de acuerdo en que el/futuro/estará lleno de sorpresas.

(After the meal we were talking about the/present/and we agreed that the/future/will be full of surprises).

(3) Durante toda la sobremesa charlamos sobre el/regalo/y estuvimos de acuerdo en que el/obsequio/fue toda una sorpresa.

(After the meal we were talking about the/gift/and we agreed that the/gift/was really surprising).

Memory demand was manipulated by changing the number of words between the target [“presente” in sentence (2) or “regalo” in sentence (3)] and the disambiguating word [“futuro” in sentence (2) or “obsequio” in sentence (3)] context (five words vs. seven words). By adding two additional words between the target and the disambiguating word, low load sentences were changed into high load sentences. Their results showed that WM load influences on-line and global comprehension in reading for translation but not for repetition in both the translators and the bilinguals. This demonstrates that experimental task may modulate WM effects in L2 syntactic processing.

Therefore, the present study aimed to explore the potential influences of WMC by using the operation span and differences in task-specific demands on processing complex sentences in L2. Experiment 1 investigated effects of WMC in processing wh-extractions via a self-paced grammatical judgment task. Experiment 2 examined whether WMC affects the processing of wh-extractions in a translation task.

As a type of complicated structure in English, one feature of the wh-extraction is that the argument of the main verb travels to another location, often a long distance from its typical location. For example, in sentence (4a), the noun phrase “the girl” is in its typical location, but in sentence (4b), it traveled to the head location of the wh-question and was substitute by the word “who” the filler. Under such circumstances, the original typical location of the noun phrase “the girl” was named the gap or trace, represented by *t*. If a parenthetical, like “the mother know” was inserted between the filler and its gap, increasing the distance between them, then a long-distance wh-filler-gap dependency (also called wh-extraction) is created, as in sentence (5). If the replaced noun phrase is the subject of the clause, then a subject extraction filler gap is formed, as in sentence (5a). If the replaced noun phrase is the object of the clause, then an object extraction filler gap is formed, as in sentence (5b).

(4a). Tom loved the girl.
(4b). Who did Tom love *t*?
(5). Who did the mother know Tom loved *t*?
(5a). Who did the mother know *t* loved the girl?
(5b). Who did the mother know the girl loved *t*?

There exists a considerable distance between the wh-word and the gap location in long-distance wh-extractions, which poses a great challenge to the parser to interpret. Thus, when parsing the wh-extraction sentence, the individual has to keep a great deal of information in WM so as to translate the dislocated wh-filler in its typical location. For L2 learners, wh-extraction might bring an additional burden on the already heavy WM load caused by the behavior of reading or listening in a L2. The wh-extraction, therefore, can serve as an ideal setting in which to explore if individual WMC provide advantages when reading in a L2 by easing access to potentially helping semantic information in syntactic reanalysis. Studies on wh-extraction comprehension found that the comprehension difficulty differed between the subject- and object-extractions (Juffs and Harrington, 1995; Dussias and Piñar, 2010; Jackson and van Hell, 2011), with some discovered that Chinese-English bilinguals are much slower in processing the complement clause (e.g., loved the girl) when processing subject-extractions (5a) compared with object-extractions (5b), suggesting that subject-extractions pose more difficulty than object-extractions.

The reason that sentence type was included in our design is that sentence complexity may be a potential factor causing the inconsistent findings on WMC and L2 syntactic processing. The structural complexity differed between subject-extractions and object-extractions. Thus, the two kinds of sentences may have different requirements of working memory, which can help elucidate the specific conditions under which working memory comes into play.

Furthermore, the current study used translation as a reading task, not just as a way to elicit output. Recent L1 studies have reported that task demands and reading goals can affect sentence processing (Swets et al., 2008). Thus, by comparing reading processes involved in grammatical judgment and translation tasks, the present study can find out if there are possible influences of reading goals on L2 sentence comprehension. Thus, the translation task in this study might help broaden our comprehension of how L2 sentence processing can be influenced by reading goals. Task influences are in addition especially informative to the issue of whether the Constrained Capacity Model can explain L2 as well as L1 sentence processing. L2 learners' performance on a translation task can indicate how they understand the input, which is useful for exploring whether L2 sentence processing can also be explained by the Constrained Capacity Model, which predicts that task or reading goals can affect language processing (Just and Carpenter, 1992; Just et al., 1996).

We predicted that WMC measured through the OSPAN task would show its effects on processing wh-extractions, and its effects on subject-extractions may be larger than that on object-extractions in the reading latencies or accuracy rate. The hypothesis is based on the findings that Chinese-English bilinguals are much slower in processing the complement clause (e.g., loved the girl) when processing subject-extractions (5a) compared with the complement clause (e.g., the girl loved) in object-extractions (5b) suggesting that subject-extractions pose more difficulty than object-extractions (Juffs and Harrington, 1995; Dussias and Piñar, 2010; Jackson and van Hell, 2011). As for the task effect, we predicted that WMC will show differential influences in the two tasks. Effects of WMC in the translation task may be larger than that in grammatical judgment in the reading latencies or accuracy rate. The cognitive theories of the translation process (Gerver, 1976; Danks and Griffin, 1997) assumed that there are three important processes occurring in translation (namely, to understand a source language, to shift between a source language and a target language, to produce in a target language). One of these theories, the horizontal approach, proposes that translation contains reformulation, which is producing semantic matches between the lexical and syntactic entries in the two languages (Gerver, 1976; Danks and Griffin, 1997). A grammatical judgment task is defined as a task in which the learners discriminate between well-formed and ill-formed sentences (Suzuki et al., 2006). Ellis (2004) proposed three potential principal processing operations involved in a grammatical judgment task: semantic processing, noticing, and metalinguistic reflection. When performing a grammatical judgment task, L2 learners first need to understand the meaning of a sentence (i.e., semantic processing). Then they attempt to find out whether something is grammatically incorrect in the sentence (i.e., noticing). Finally, L2 learners may reflect on what is incorrect in the sentence and why it is so (i.e., metalinguistic reflection). L2 learners may potentially undertake all three operations and thus draw on explicit knowledge in performing grammatical judgment tasks. Comparatively speaking, the translation process requires additional attention and increased cognitive load compared to a grammatical judgment task, because one needs to establish semantic and syntactic matches between two languages during translation in a parallel manner (Gerver, 1976; Danks and Griffin, 1997). Therefore, complex processes are involved in translation and thus impose a larger demand on the working memory.

## Experiment 1

### Method

#### Participants

Participants were 50 Chinese-English bilinguals (19 male), between 19 and 26 years old (*M* = 22.96, *SD* = 1.51) who were students at Beijing Normal University and who had learned English in middle school after age 12. The participants finished the grammatical judgment task first, and then the Operation Span (OSPAN) was used to measure their WMC. The language background measures were all administered last.

The bilingual participants were proficient in English, with Test of English as a Foreign Language (TOEFL) scores >90, combined Graduate Record Examination (GRE) scores higher than 1,200, or a admissible score on the Test for English Majors (TEM) of 8 in the study year. The participants self-rate their listening, speaking, reading, and writing English skills on a 6-point scale, with 1 being not fluent and 6 being high fluent. The scores of their English proficiency were acquired by the Oxford placement test (OPT). There were 25 multiple-choice questions and a cloze test in the OPT, with a maximum score of 50. The mean self-rating and OPT scores of the participants in Experiments 1 and 2 are presented in Table 1. The participants had an intermediate L2 proficiency.

**Table 1:** **Means on English proficiency rating and test (*SD*s) in both Experiment 1 and 2**.

	**Listening**	**Speaking**	**Reading**	**Writing**	**Opt**
Experiment1	4.4 (0.96)	4.0 (0.62)	3.9 (0.93)	3.9 (0.88)	41.6 (2.53)
Experiment2	4.4 (0.93)	4.0 (0.68)	3.8 (0.93)	3.9 (0.99)	41.5 (2.68)

*OPT, Oxford placement test*.

#### Working memory measure

The Operation Span (OSPAN) was used to measure the WMC (Conway et al., 2005). Unsworth et al. have shown that the OSPAN was well-correlated with other measures of WMC, and its internal consistency (alpha = 0.78) and test-retest reliability (0.83) were both good enough. The measure of OSPAN was computer-based, with participant responses made with mouse clicks (Unsworth et al., 2005). The task comprised three practice sessions followed by a formal session. The first practice session was a simple letter span task. Participants saw a series of letters appear on the screen, one at a time, and then were prompted to recall them in the same order as presented. Responses were made by selecting the appropriate letters from an array of the alphabet on the screen; no verbal response was required. Feedback about the number of correctly recalled letters was provided by the computer program following each trial. The second practice session was a math operations task. First, a math operation [e.g., (1^*^2) + 1 = ?] appeared on the screen. After performing the calculation, the participant clicked the mouse to move to the next screen. A number (e.g., “3”) appeared, and the participant was asked to note whether the number was the correct answer to the math operation by clicking either “True” or “False.” Again, accuracy feedback was given after each trial. The math operation practice was used in order to familiarize participants with the math part of the experiment, but also to estimate how long it took each participant to solve the math problems. This estimate can account for individual differences in math problem-solving speed. The response time measured for the math operation part of the OSPAN task refers to the time between onset of the equation to the participant clicking to advance to the next screen. After the math practice section, the program calculated each individual's mean time required to solve the equations. This mean response time (plus 2.5 standard deviations) was then used as a time limit for the math portion of the formal session. The third practice session required participants to perform combined math operations and letter recall. Participants first saw a math operation and then a letter to be recalled. Calculation of the math operation had to be performed between the letter presentation and the recall of the letters. If it took the participants more time to solve the math problem than their average time plus 2.5 *SD*, then the program automatically moved on. This served to prevent participants from rehearsing the letters when they should be devoting cognitive resources to the calculations. After the participant finished all practice sessions, the program proceeded into to the formal session. Participants performed math operation and letter recall tasks. There were 75 letter recall trials with 75 math operations, with three sets each of set sizes ranging from 3 to 7 (9 + 12 + 15 + 18 + 21 = 75). Cumulative accuracy for the math task was displayed in red font in the upper right-hand corner of the screen during letter recall; participants were instructed to maintain math operation accuracy at or above 85% throughout the session. The OSPAN score was the sum of the sizes of all perfectly recalled letter sets. For instance, if an individual correctly recalled 3 letters in a set size of 3, 4 letters in a set size of 4, and 4 letters in a set size of 5, their OSPAN score would be 7 (3 + 4 + 0).

A median split at 53 of the OSPAN score was performed. Following the study of Hestvik et al. (2012) and also that of Roberts et al. (2007), we assigned subjects to the low span group if their span was less than or equal to the median of the group's operation span, and to the high span group if it was greater than the median. As a result, there were 25 high span subjects (*M* = 64.06, *SD* = 5.24) and 25 low span subjects (*M* = 38.20, *SD* = 8.27). The two groups have no differences in their self-reported English listening [4.36 (*SD* = 0.86) vs. 4.35 (*SD* = 0.98), *p* = 0.34], speaking [4.16 (*SD* = 0.55) vs. 3.96 (*SD* = 0.66), *p* = 0.11], reading [3.92 (*SD* = 0.64) vs. 4.03 (*SD* = 0.92), *p* = 0.91] and writing abilities [3.84 (*SD* = 0.94) vs. 3.77 (*SD* = 1.03), *p* = 0.47], nor in their Oxford Placement Test scores [40.96 (*SD* = 2.37) vs. 41.31(*SD* = 2.59), *p* = 0.85].

#### Experimental materials

There were 32 pairs of target stimuli in this experiment. The wh-questions were used as target stimuli in which the beginning wh-filler was either the subject or the direct object of the complement clause, as in sentences (6) and (7).

(6) Who do you think loved the comedian with all his heart? (subject-extraction)
                  region1 region2
(7) Who do you think the comedian loved with all his heart? (object-extraction)
                  region1 region2

Whether the initial wh-filler was the subject or the direct object of the complement clause was disambiguated via word order. Each target sentence also included a prepositional phrase after the critical region, in order to guarantee that the critical region did not coincide with the end of the sentence. In the initial clause (e.g., *Who do you think…*), we relied on three main verbs, namely, “think” “say” and “suspect.” These three verbs were chosen from the larger set of verbs used by Dussias and Piñar (2010), as well as Juffs and Harrington (1995).

The subject- and object-extractions are disambiguated via word order in English, thus, the second critical region for the reaction time analysis was defined as the verb and noun phrase in the complement clause [e.g., *loved the comedian* or *the comedian loved* in examples (6) and (7)]. Reading times on these three words were averaged to obtain the reading time for the second critical region (Dussias and Piñar, 2010; Jackson and van Hell, 2011). In addition to region 2, the main verb in both the subject- and object-extractions [e.g., *think* in examples (6) and (7)] was defined as region 1, so as to make certain that there were no differences in reaction times before the critical region.

Besides the 32 pairs of experimental stimuli, subjects read 32 grammatical fillers and 64 ungrammatical fillers. There are some other grammatical and ungrammatical wh-questions (e.g., *Who does Emily want to invite to her party?*) as well as declarative sentences in the 96 filler items. We divided the 32 pairs of target stimuli into two lists, so that subjects read over 16 subject-extractions and 16 object-extractions, but they did not read more than one version of each target stimuli. The 32 target sentences were presented in a randomized order along with 32 grammatical filler items and the 64 ungrammatical fillers. Thus, there were an equal number of grammatical and ungrammatical sentences.

#### Design

We used a 2 (WMC: high, low) × 2 (sentence type: subject-extraction, object-extraction) mixed design. WMC was a between-subjects factor, and sentence type was a within-subjects factor.

#### Procedure

Participants completed the task individually in a silent room. They were seated in front of a computer screen. The target and filler items were presented using the self-paced reading paradigm (Just et al., 1982) via E-Prime 1.1. Participants first received written instructions. In keeping with previous studies (Jackson and Dussias, 2009; Jackson and Bobb, 2009), noun phrases, adverbial phrases, and prepositional phrases were presented entirely, while all other words were presented in a word-by-word fashion. Each trial started with the word “READY” appearing in the center of the screen. At this moment, subjects could push the space bar to start reading the sentence. The fixation word was cleared away, and the beginning word or phrase of the sentence emerged, left justified on the screen. When subjects pushed the space bar again, the first word or phrase vanished and the following word or phrase emerged. Participants were asked to read over each word or phrase quietly and to push the spacebar to present each successive word or phrase on the computer through to the end of each sentence. The time between the appearance of each word or phrase and pressing the space bar was recorded. Similar to previous studies examining this type of wh-question, when participants finished reading each sentence, a prompt appeared on the screen that asked them to judge whether the sentence they had just read was grammatical or ungrammatical. They responded on the keyboard by pressing J for “yes” and F for “no.” Prior to the formal experiment, each participant had 12 practice trials.

### Results

The reaction time analysis only included results from correctly judged items. The data from incorrect responses were excluded, this resulted in the exclusion of 4.4% of the data. Mean accuracy rates for the sentences are presented in Table 2.

**Table 2:** **Mean accuracy rates (%) (*SD*s) of subject/object extractions of Experiment 1**.

**WMC**	**Sentence type**	
	**Subject-extractions**	**Object-extractions**
High	87 (0.11)	77 (0.20)
Low	87 (0.13)	80 (0.16)

*WMC, Working memory capacity*.

A two-way analysis of variance was conducted both by subjects (F1) and by items (F2) on the accuracy rates. Data analysis on the accuracy rates showed that the main effect of sentence type was significant, *F*_1__(1, 48)_ = 9.94, *MSE* = 0.19, *p* = 0.003, η^2^ = .17; *F*_2__(1, 30)_ = 16.27, *MSE* = 0.12, *p* < 0.001, η^2^ = 0.35. Participants were significantly more accurate in the subject-extractions than the object-extractions. There was not a significant main effect of WMC, *F*_1__(1, 48)_ = 0.19, *MSE* = 0.01, *p* = 0.67, η^2^ = 0.00; *F*_2__(1, 30)_ = 0.19, *MSE* = 0.04, *p* = 0.67, η^2^ = 0.01. Furthermore, the interaction between sentence type and WMC was not significant either, *F*_1__(1, 48)_ = 0.29, *MSE* = 0.07, *p* = 0.59, η^2^ = 0.01; *F*_2__(1, 30)_ = 0.48, *MSE* = 0.04, *p* = 0.5, η^2^ = 0.02. Moreover, we also explored the relationship between the participant's WMC and their mean accuracies in the sentences using partial correlation with OPT as a covariate. Correlational analysis showed that the WMC was not correlated with the participants' mean accuracies (*r* = 0.11, *p* = 0.42).

Mean reactions times for the two critical sentence regions are presented in Table 3.

**Table 3:** **Mean response times (ms) (*SD*s) of the critical regions in subject/object extractions of Experiment 1**.

**WMC**	**Sentence type**			
	**Subject-extractions**		**Object-extractions**	
	**Region 1**	**Region 2**	**Region 1**	**Region 2**
High	833 (417)	1,534 (478)	770 (367)	1,515 (467)
Low	821 (273)	1,749 (431)	807 (347)	1,771 (545)

*WMC, Working memory capacity*.

#### Region 1

Data analysis on the mean reading times showed that there was not a significant main effect of WMC, *F*_1__(1, 48)_ = 0.03, *MSE* = 5005.26, *p* = 0.87, η^2^ = 0.00; *F*_2__(1, 30)_ = 0.01, *MSE* = 1184.25, *p* = 0.91, η^2^ = 0.00. The main effect of sentence type was not significant either, *F*_1__(1, 48)_ = 0.91, *MSE* = 29892.56, *p* = 0.34, η^2^ = 0.02; *F*_2__(1, 30)_ = 0.71, *MSE* = 17547.94, *p* = 0.41, η^2^ = 0.02. The interaction between sentence type and WMC was not significant either, *F*_1__(1, 48)_ = 0.34, *MSE* = 10983.46, *p* = 0.57, η^2^ = 0.01; *F*_2__(1, 30)_ = 0.01, *MSE* = 151.89, *p* = 0.94, η^2^ = 0.00.

#### Region 2

Data analysis showed that the main effect of WMC was significant, *F*_1__(1, 48)_ = 3.51, *MSE* = 1386371.31, *p* = 0.07, η^2^ = 0.07; *F*_2__(1, 30)_ = 9.92, *MSE* = 1053101.85, *p* = 0.004, η^2^ = 0.25, indicating that reaction times in the high WMC group were faster than those in the low WMC group. There was not a significant main effect of sentence type, *F*_1__(1, 48)_ = 0.01, *MSE* = 50.21, *p* = 0.98, η^2^ = 0.00; *F*_2__(1, 30)_ = 0.12, *MSE* = 3919.74, *p* = 0.74, η^2^ = 0.00. The interaction between sentence type and WMC also did not reach significance, *F*_1__(1, 48)_ = 0.16, *MSE* = 10812.18, *p* = 0.7, η^2^ = 0.00; *F*_2__(1, 30)_ = 0.98, *MSE* = 33151.29, *p* = 0.33, η^2^ = 0.03.

Moreover, we also examined the relationship between the participant's WMC and their mean reading times for the two critical regions in the sentences using partial correlation with OPT as a covariate. Correlational analysis showed that the WMC was negatively correlated with participants' mean reading times marginally in region 2 (*r* = −0.26, *p* = 0.07), but not with region 1 (*r* = 0.02, *p* = 0.89).

In Experiment 1, Chinese-English bilinguals showed WMC effects during grammatical judgment of wh-questions only with respect to reading latencies but not to accuracy. Participants with high WMC were much faster in processing wh-extractions than those with low WMC, which suggests that WMC affects L2 syntactic processing. In Experiment 2 we used a translation task in order to further compare whether a different experimental task modulates the effects of WMC during processing of wh-extractions.

## Experiment 2

### Methods

#### Participants

Participants were 50 Chinese-English bilinguals (14 male) between 18 and 33 years old (*M* = 23, *SD* = 3) who were students at Beijing Normal University and who learned English in middle school after age 12. None of them had participated in Experiment 1. We used the same standards as those in Experiment 1 to select the participants. Participants' listening, speaking, reading, and writing English skills were assessed in a same way to that of Experiment 1. The mean self-rating and OPT scores of the participants in Experiment 2 are presented in Table 1.

The two groups of participants in Experiments 1 and 2 have no differences in their self-rated English listening (4.36 vs. 4.37, *p* = 0.68), speaking (4.02 vs. 4, *p* = 0.7), reading (3.9 vs. 3.81, *p* = 0.99), or writing abilities (3.86 vs. 3.92, *p* = 0.7), nor in their OPT scores (41.66 vs. 41.52, *p* = 0.73). The measurement of WMC was the same as in Experiment 1. The median operation span of all the subjects was 56.5. As in Experiment 1, subjects were assigned to the low-span group if their span was less than or equal to the median of the group's operation span, and to the high span group if it was larger than the median. As a consequence, there were 25 high-span participants (*M* = 66.16, *SD* = 4.94) and 25 low-span participants (*M* = 39.6, *SD* = 9.2). The two span groups have no differences in their self-rated English listening [4.32 (*SD* = 0.9) vs. 4.48 (*SD* = 0.96), *p* = 0.76], speaking [3.96 (*SD* = 0.68) vs. 4.08 (*SD* = 0.7), *p* = 0.38], reading [3.8 (*SD* = 0.87) vs. 3.88 (*SD* = 1.01), *p* = 0.71], or writing abilities [3.88 (*SD* = 1.09) vs. 3.96 (*SD* = 0.89), *p* = 0.77], nor in their OPT scores [40.92 (*SD* = 2.96) vs. 40.08 (*SD* = 2.29, *p* = 0.12)].

#### Materials

The 32 pairs of target stimuli were the same as those in Experiment 1. Additionally, 64 filler items were presented. These 64 fillers included additional wh-extractions, subject clauses, object clauses, adverbial clauses, and appositive clauses. We divided the 32 pairs of target sentence into two lists. In such a way, the subjects translated 16 subject-extractions and 16 object-extractions, but they did not translate more than one version of each target stimuli. The 32 target sentences were presented in a randomized order along with 64 grammatical filler items. The critical region for the reading time analyses was the same as in Experiment 1.

#### Design

We also used a 2 (WMC: high, low) × 2 (sentence type: subject-extraction, object-extraction) mixed design. WMC was a between-subjects factor, and sentence type was a within-subjects factor.

#### Procedure

The procedure of the translation task was the same as the grammatical judgment, apart from that the subjects have to translate the target (L2) sentence they just read into Chinese verbally. The target and filler items were also presented using the self-paced-reading paradigm. In keeping with Experiment 1, noun phrases, adverbial phrases, and prepositional phrases were also presented wholly, but all the other words were presented in a word-by-word fashion. Similar to Experiment 1, when subjects read over every sentence, a prompt would emerge on the computer screen that asked them to translate the target sentence they just read into Chinese. Translations were recorded via a digital voice recorder for analysis. Participants also translated 12 practice stimuli before the formal experiment to familiar with the task.

### Results

The translations of subjects were transliterated by the experimenter and coded as subject-extraction-correct, subject-extraction-incorrect, object-extraction-correct, and object-extraction-incorrect (where “correct” stands for thematic roles were correctly allotted and “incorrect” stands for thematic roles were interpreted in an inverted order from the original). The coder was a graduate student who majored in English and could ensure the accuracy of coding.

Only results from items correctly translated were included in the reading time analyses. Incorrect responses were excluded (4.90%). Additionally, there were inappropriate translations where the participants did not produce any subject- or object-extraction structures, or they did not comprehend the input. All these were treated as outliers and were removed from further analysis (6.35%). Mean translation accuracy rates for the sentences are presented in Table 4.

**Table 4:** **Mean accuracy rates (%) (*SD*s) of subject/object extractions of Experiment 2**.

**WMC**	**Sentence type**	
	**Subject-extractions**	**Object-extractions**
High	91 (0.07)	85 (0.09)
Low	87 (0.11)	75 (0.14)

*WMC, Working memory capacity*.

Statistical analysis demonstrated that there was a significant main effects of sentence type, *F*_1__(1, 48)_ = 40.93, *MSE* = 0.22, *p* < 0.001, η^2^ = 0.46; *F*_2__(1, 30)_ = 21.21, *MSE* = 0.14, *p* < 0.001, η^2^ = 0.41. Subjects were significantly more accurate in judging the subject- than the object-extractions. The main effect of WMC was also significant, *F*_1__(1, 48)_ = 6.81, *MSE* = 0.12, *p* = 0.01, η^2^ = 0.12; *F*_2__(1, 30)_ = 5.5, *MSE* = 0.08, *p* = 0.03, η^2^ = 0.16, with high WMC participants more accurate in processing wh-extractions than low WMC subjects. The interaction between sentence type and WMC was not significant, *F*_1__(1, 48)_ = 3.85, *MSE* = 0.02, *p* = 0.06, η^2^ = 0.07; *F*_2__(1, 30)_ = 1.99, *MSE* = 0.01, *p* = 0.17, η^2^ = 0.06. The partial correlations with OPT as a covariate showed that the WMC was not significantly correlated with the mean accuracy rates (*r* = 0.03, *p* = 0.84).

Mean reading times for the critical sentence regions are presented in Table 5.

**Table 5:** **Mean response times (ms) (*SD*s) of the critical regions in subject/object extractions of Experiment 2**.

**WMC**	**Sentence type**			
	**Subject-extractions**		**Object-extractions**	
	**Region1**	**Region2**	**Region1**	**Region2**
High	635 (294)	1,390 (396)	619 (293)	1,420 (481)
Low	630 (139)	1,453 (457)	699 (231)	1,375 (634)

*WMC, Working memory capacity*.

#### Region 1

There were no significant main effects of sentence type, *F*_1__(1, 48)_ = 1.41, *MSE* = 18043.5, *p* = 0.24, η^2^ = 0.03; *F*_2__(1, 30)_ = 1.88, *MSE* = 16852.61, *p* = 0.18, η^2^ = 0.06, and neither of WMC, *F*_1__(1, 48)_ = 0.32, *MSE* = 35525.45, *p* = 0.57, η^2^ = 0.01; *F*_2__(1, 30)_ = 0.46, *MSE* = 22601.52, *p* = 0.5, η^2^ = 0.02. The interaction between sentence type and WMC did not reach significance either, *F*_1__(1, 48)_ = 3.59, *MSE* = 46143.12, *p* = 0.07, η^2^ = 0.07; *F*_2__(1, 30)_ = 3.42, *MSE* = 30698.66, *p* = 0.07, η^2^ = 0.1.

#### Region 2

There were no significant main effects of sentence type, *F*_1__(1, 48)_ = 0.3, *MSE* = 14123.95, *p* = 0.59, η^2^ = 0.01; *F*_2__(1, 30)_ = 0.01, *MSE* = 350.17, *p* = 0.92, η^2^ = 0.00, and neither of WMC, *F*_1__(1, 48)_ = 0.04, *MSE* = 1958.29, *p* = 0.95, η^2^ = 0.00; *F*_2__(1, 30)_ = 0.09, *MSE* = 22279.17, *p* = 0.77, η^2^ = 0.00. The interaction between sentence type and WMC was not significant either, *F*_1__(1, 48)_ = 1.54, *MSE* = 73689.56, *p* = 0.22, η^2^ = 0.03; *F*_2__(1, 30)_ = 0.47, *MSE* = 14458.11, *p* = 0.50, η^2^ = 0.02.

The partial correlations with OPT as a covariate showed that the WMC was not significantly correlated with the reading times in both region 1 (*r* = 0.004, *p* = 0.98) and region 2 (*r* = −0.08, *p* = 0.58).

Unlike Experiment 1, Experiment 2 showed that WMC only affects the accuracy rates but not the reading times in processing wh-extractions in a translation task. Participants with a high WMC are more accurate in processing wh-extractions compared with those with low WMC.

## Discussion

In the present study, two experiments were conducted to explore the potential influences of WMC, as measured by an OSPAN task, and differences in task-specific demands on the processing of second language sentence. We investigated processing of wh-extractions in L2 learners with different WMC using both a grammatical judgment task and a translation task. Results showed significant effects of WMC in both the reading latencies during the grammaticality judgment and accuracy rates in the translation task. In addition, participants were more accurate in the subject extractions than in the object extractions in both the grammatical judgment and the translation tasks.

Most of the previous L2 studies used the reading span task, especially L2 reading, to measure the participants' WMC. No matter the assessment language, both L1 and L2 reading span tasks require high levels of cognitive processing plus prior knowledge of the language and subject matter. There is an unavoidable confound of linking WMC and language processing ability in this task. The OSPAN is less sensitive to influences of reading or language ability and more likely to reflect bilinguals' genuine WMC (Sanchez et al., 2010). Although the OSPAN uses less language-specific content relative to reading span, it still uses more than symmetry span or counting span. Participants need to complete math problems using Arabic (English) numerals, respond true or false in English, and remember letters from the English alphabet. In this way, language comfort might be predicting OSPAN to some extent. Our participants would have familiarity with Arabic numerals and alphabetic letters from their use in Chinese writing and Pinyin, as well as English L2 instruction. Moreover, individual variation in the knowledge of English alphabets is likely much smaller than overall English (L2) proficiency. So, the effect of language comfort is controlled as much as possible with the OSPAN task. Therefore, in the present study, OSPAN was used to measure the WMC of Chinese-English bilinguals. We found the effects of WMC in both experiments, providing some evidence for WMC effects in L2 sentence processing irrespective of language proficiency.

The present study found effects of WMC only in the reading latencies but not in accuracy rates during the grammaticality judgment task. But in the translation task, effects of WMC were only found in the accuracy rates but not in response latencies. Such results are consistent with the Constrained Capacity Model, in that the allocation of cognitive resources was sensitive to the specific tasks employed. It is possible that in a grammatical judgment task, L2 learners have to process each element of every sentence carefully and check for possible grammatical mistakes until the end of the whole sentence, which imposes a great demand of cognitive resources and consumes lots of reading time. In such a situation, participants with a high WMC can process and store more information throughout the task than participants with low WMC. As a result, an effect of WMC was observed in reading latencies in the grammatical judgment task in Experiment 1. Although, we predicted that the effects of WMC in the translation task would be larger than that in grammatical judgment, the current results provide no support for this prediction. By comparing the data in Tables 3 and 5, we found that the response times in the grammatical judgment were longer than the response time in the translation task. It is possible that the participants in the translation task read each word in the whole sentence quickly so as to get the meaning of the whole sentence more quickly, thus they did not engage in detailed syntactic processing of words. This would make their response times much shorter, diminishing any temporal effects of WMC. Yet participants with high WMC can keep the meaning of the just read sentences in their memory much better, therefore they showed advantages in the accuracy of translation compared with the participants with low WMC.

Previous studies have found that the processing of subject extractions is harder than that of object extractions, as is seen in both the accuracy and response times (Juffs and Harrington, 1995; Dussias and Piñar, 2010; Jackson and van Hell, 2011). While, the current study found that participants were more accurate in subject extractions than in object extractions in both the grammatical judgment and translation tasks, they processed subject extractions and object extractions similarly in their response times for the critical regions. The reasons for these inconsistent results await further research. The present study also found that the effects of WMC were not modulated by sentence type. Because the interaction between WMC and sentence type was not found in the current study, this means that the effect of WMC was the same in both the subject- and object-extractions. We hope that future work can explore the difficulties posed by subject- and object-extractions in L2 learners of various languages, and also compare how WMC affects the processing of these two types of complex structures.

The limitations of the current study were as following. Firstly, the participants in the present study are intermediate proficient, but we do not know if the same results can be obtained with high proficient Chinese-English bilinguals. Therefore, the current findings are only limited to intermediate proficient participants. Secondly, in Experiment 2, the WMC was not correlated with participants' mean accuracies after the effect of L2 proficiency was partial removed. Such results indicated that the effect of WMC was possibly related to the L2 proficiency in Experiment 2. Therefore, it is necessary to further explore the effect of WMC on complex sentences with high proficient bilinguals.

To summarize, the present study differs from previous studies in that it explores the role of WMC in L2 sentence processing by using the operation span. We also examined whether effects of WMC are modulated by experimental tasks. The results showed that participants with high WMC are much faster in the grammatical judgment and more accurate in translation of wh-extractions, providing a much better understanding of the boundary conditions of WMC effects. Although the current study only found a weak effect of WMC, it broadens our understanding of how L2 processing can be modulated by reading goals and tasks, and showed that the Constrained Capacity Model can account for L2 processing as well.

## Ethics statement

The Ethics Committee of the School of Psychology, Beijing Normal University.

## Author contributions

BC and HZ together initiated the design for this paper. HZ collected the data, undertook the statistical analysis and wrote the paper. SR made critical comments and revision on the manuscript. BC is the principal investigator of this project, and supervised the statistical analysis and the manuscript writing and revision.

## Funding

Supported by the Scientific Foundation of Institute of Psychology, Chinese Academy of Sciences, No.Y6CX242007.

### Conflict of interest statement

The authors declare that the research was conducted in the absence of any commercial or financial relationships that could be construed as a potential conflict of interest.
